# Crystal structure of the bis­(cyclo­hexyl­ammonium) succinate succinic acid salt adduct

**DOI:** 10.1107/S2056989015012621

**Published:** 2015-07-08

**Authors:** Modou Sarr, Aminata Diasse-Sarr, Libasse Diop, Laurent Plasseraud, Hélène Cattey

**Affiliations:** a Laboratoire de Chimie Minérale et Analytique (LACHIMIA), Département de Chimie, Faculté des Sciences et Techniques, Université Cheikh Anta Diop, Dakar, Senégal; b ICMUB UMR 6302, Université de Bourgogne Franche-Comté, Faculté des Sciences, 9 avenue Alain Savary, 21000 Dijon, France

**Keywords:** crystal structure, organic salt, mol­ecular adduct, hydrogen bonds, succinate, succinic acid, cyclo­hexyl­ammonium cation

## Abstract

The crystal structure of the title salt adduct, 2C_6_H_14_N^+^·C_4_H_4_O_4_^2−^·C_4_H_6_O_4_, consists of two cyclo­hexyl­ammonium cations, one succcinate dianion and one neutral succinic acid mol­ecule. Succinate dianions and succinic acid mol­ecules are self-assembled head-to-tail through O—H⋯O hydrogen bonds and adopt a *syn*–*syn* configuration, leading to a strand-like arrangement along [101]. The cyclo­hexyl­ammonium cations have a chair conformation and act as multidentate hydrogen-bond donors linking adjacent strands through inter­molecular N—H⋯O inter­actions to both the succinate and the succinic acid components. This results in two-dimensional supra­molecular layered structures lying parallel to (010).

## Abstract 2

The title salt adduct comprises two cyclo­hexyl­ammonium cations, one succinate anion and one mol­ecule of succinic acid, linked together through inter­molecular hydrogen-bonding inter­actions giving a two-dimensional layer-like self-assembly lying parallel to (010).

## Chemical context   

In the field of crystal engineering, di­carb­oxy­lic acids constitute very suitable building blocks which can act as polydirectional synthons and thus present numerous possibilities for mol­ecular assembly through the formation of hydrogen-bonded networks (Ivasenko & Perepichka, 2011 ▸). Furthermore, the additional involvement of amines, *via* the formation of ammonium cations, significantly increases the potential for linkage and the topological diversity (Yuge *et al.*, 2008 ▸; Lemmerer, 2011 ▸). Some papers dealing with spectroscopic studies on quaternary ammonium hydrogenoxalates have been reported from our laboratory (Gueye & Diop, 1995 ▸). In the scope of our current studies on the inter­actions between quaternary ammonium salts of carb­oxy­lic acids and halogenidotin(IV) complexes (Gueye *et al.*, 2014 ▸), the reaction involving cyclo­hexyl­amine and succinic acid was initiated and led to the isolation of the title organic salt adduct 2C_6_H_14_N^+^·C_4_H_4_O_4_^2−^·C_4_H_6_O_4_, (I), the structure of which is reported herein.
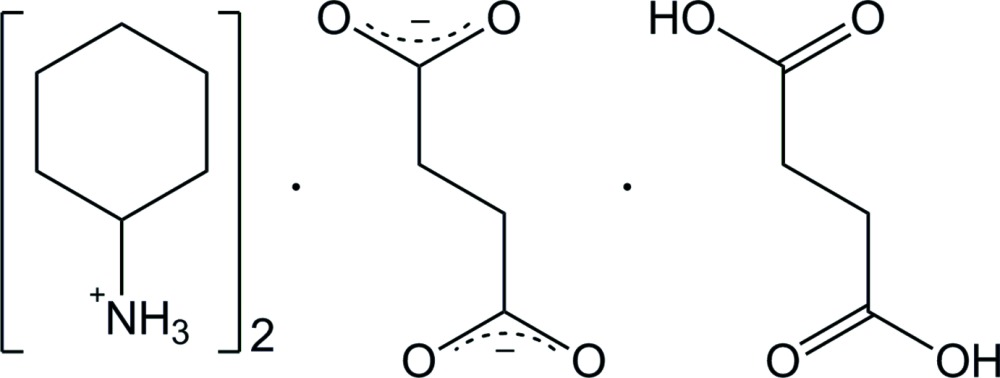


## Structural comments   

**Figure 1:**
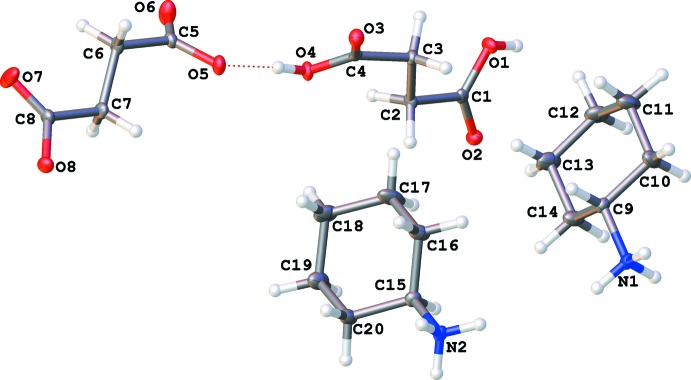
A view of the two cyclo­hexa­minum cations, the succinate dianion and the succinic acid adduct species in the asymmetric unit of (I), showing the atom labeling. Displacement ellipsoids are drawn at the 50% probability level.

**Figure 2:**
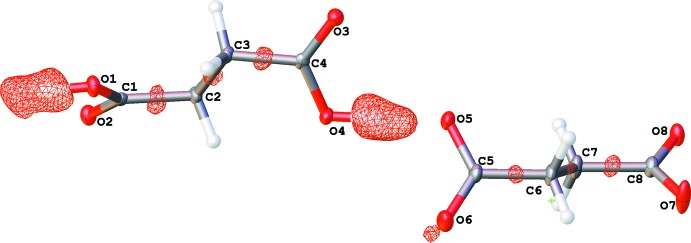
Electron-density mapping around C_4_H_6_O_4_ and C_4_H_4_O_4_^2−^, showing the precise location of acidic protons.

The asymmetric unit of (I) contains two cyclo­hexyl­ammonium cations, one succinate dianion and one mol­ecule of succinic acid (Fig. 1 ▸). By comparison with previous examples (Büyükgüngör & Odabas˛ogˇlu, 2002 ▸; Bruno *et al.*, 2004 ▸; Du *et al.*, 2009 ▸; Zhang *et al.*, 2011 ▸; Froschauer & Weil, 2012 ▸), it is inter­esting to note that the carbon–oxygen bond distances recorded for the succinic acid [C1—O1 = 1.2974 (17), C1—O2 = 1.2356 (17), C4—O3 = 1.2367 (17), C4—O4 = 1.2961 (16)] and the succinate dianion [C5—O5 = 1.2955 (17), C5—O6 = 1.2356 (18), C8—O7 = 1.2348 (18) and C8—O8 = 1.2894 (17)] are very similar. In general, a more pronounced difference in length is expected between the C=O bond and the C—OH bond of succinic acid (in the range of 0.1 Å), while for the succinate dianion the deviation between the C—O bonds is narrowed (in the range of 0.01 Å). Thus, to confirm more accurately the nature of the components of (I), namely the presence of distinct succinic acid and succinate species, electron-density mapping has been performed (Fig. 2 ▸). It follows that the location of the acidic protons is clearly established, confirming unambiguously the composition of (I). Moreover, the relative equalizing of the carbon–oxygen bonds can be explained by the contribution of concomitant N—H⋯O inter­actions involving all oxygen atoms of succinic acid and the succinate dianion with surrounding cyclo­hexyl­ammonium cations. The average C—C—C—O torsion angle, calculated on 616 succinic acids, is equal to 171 (12)° with a deviation of the mean equal to 0.4°, whereas the average torsion angle calculated on 964 succinate acids is equal to 167 (12)° with a deviation of the mean also equal to 0.4°. These results match the torsion angles found in (I) for succinic acid: 154.09 (16), 156.32 (12), 159.25 (17) and 161.07 (12)° but those found for the succinate anion are rather different: 121.41 (15), 121.78 (17), 151.8 (2) and 152.14 (13)°.

## Supra­molecular features   

**Figure 3:**
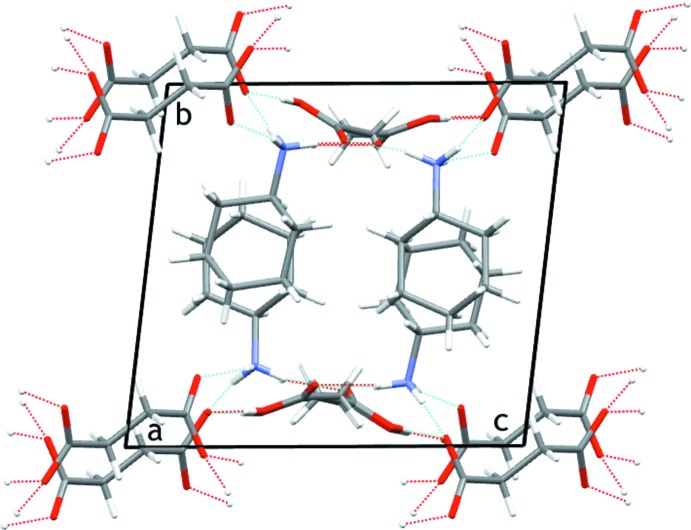
Crystal packing of (I) viewed along the *a* axis, showing the infinite strands based on succinate–succinic acid hydrogen-bonding inter­actions and linked through the cyclo­hexyl­ammoninum cations into sheets. Inter­molecular hydrogen bonds are shown as dashed blue lines. H atoms not involved in hydrogen bonding are omitted for clarity. Colour code: C dark grey, H light grey, O red, N blue.

From a supra­molecular point of view, the four components of (I) are involved in the self-assembly. The succinate dianion and succinic acid are linked head-to-tail through short O—H⋯O hydrogen bonds [2.4636 (13) and 2.4734 (13) Å] (Table 1 ▸) leading to infinite strands which extend along [101]. These inter­molecular distances are consistent with the mean of 2.52 Å with a sample standard deviation of 0.06 Å observed on a sample of 25 observations from the CSD on a set of structures containing both a succinic acid and a succinate anion. The cyclo­hexyl­ammonium cations operate as multidentate hydrogen-bond donors through N—H⋯O inter­actions linking the succinate–succinic acid strands, giving two-dimensional supra­molecular layers lying parallel to (010) (Fig. 3 ▸).

## Synthesis and crystallization   

The title compound was obtained by reacting cyclo­hexyl­amine (5.76 mL) with succinic acid (5.0 g) in a molar ratio of 2:1, in 50 mL of water, at 298 K. The resulting clear solution was allowed to evaporate at 298 K leading after a few days to colourless block-like crystals suitable for an X-ray crystal structure determination.

## Refinement   

Crystal data, data collection and structure refinement details are summarized in Table 2 ▸. All H atoms, on carbon, oxygen and nitro­gen atoms were placed at calculated positions using a riding model with C—H = 1.00 (methine) or 0.99 Å (methyl­ene) and with *U*_iso_(H) = 1.2*U*_eq_(C), or O—H = 0.84 Å (hydrox­yl), N—H = 0.91 Å (amine) with *U*_iso_(H) = 1.5*U*_eq_(O or N).

## Supplementary Material

Crystal structure: contains datablock(s) global, I. DOI: 10.1107/S2056989015012621/zs2336sup1.cif

Structure factors: contains datablock(s) I. DOI: 10.1107/S2056989015012621/zs2336Isup2.hkl

Supporting information file. DOI: 10.1107/S2056989015012621/zs2336Isup3.cml

CCDC reference: 1409738

Additional supporting information:  crystallographic information; 3D view; checkCIF report

## Figures and Tables

**Table 1:** Hydrogen-bond geometry (, )

*D*H*A*	*D*H	H*A*	*D* *A*	*D*H*A*
N1H1*A*O5^i^	0.91	1.99	2.8923(16)	173
N1H1*B*O2^ii^	0.91	2.10	2.8969(16)	146
N1H1*C*O7^iii^	0.91	1.86	2.7279(15)	158
N2H2*A*O8^iv^	0.91	2.00	2.8746(16)	160
N2H2*B*O3^i^	0.91	2.17	2.9098(15)	138
N2H2*C*O6^v^	0.91	1.94	2.7485(15)	148
O1H1O8^vi^	0.84	1.64	2.4734(13)	175
O4H4O5	0.84	1.63	2.4636(13)	175

Symmetry codes: (i) 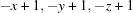; (ii) 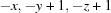; (iii) 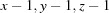; (iv) 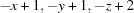; (v) 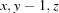; (vi) 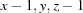.

**Table 2:** Experimental details

Crystal data	
Chemical formula	2C_6_H_14_N^+^C_4_H_4_O_4_^2^C_4_H_6_O_4_
*M*_r_	434.52
Crystal system, space group	Triclinic, *P* 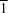
Temperature (K)	115
*a*, *b*, *c* ()	9.5147(5), 10.4479(6), 11.4082(6)
, , ()	96.789(2), 93.287(2), 90.945(2)
*V* (^3^)	1123.96(11)
*Z*	2
Radiation type	Mo *K*_1_
(mm^1^)	0.10
Crystal size (mm)	0.5 0.3 0.25

Data collection	
Diffractometer	Nonius Kappa APEXII
Absorption correction	Multi-scan (*SADABS*; Bruker, 2014 ▸)
*T*_min_, *T*_max_	0.710, 0.746
No. of measured, independent and observed [*I* > 2(*I*)] reflections	30513, 5190, 4273
*R*_int_	0.030
(sin /)_max_ (^1^)	0.652

Refinement	
*R*[*F* ^2^ > 2(*F* ^2^)], *wR*(*F* ^2^), *S*	0.043, 0.115, 1.03
No. of reflections	5190
No. of parameters	275
H-atom treatment	H-atom parameters constrained
_max_, _min_ (e ^3^)	0.38, 0.52

Computer programs: *APEX2* and *SAINT* (Bruker, 2014 ▸), *SHELXS2014* (Sheldrick, 2008 ▸), *SHELXL2014* (Sheldrick, 2015 ▸), *OLEX2* (Dolomanov *et al.*, 2009 ▸) and *Mercury* (Macrae *et al.*, 2008 ▸).

## supplementary crystallographic information

### Crystal data

**Table:**

2C_6_H_14_N^+^·C_4_H_4_O_4_^2^^−^·C_4_H_6_O_4_	*Z* = 2
*M**_r_* = 434.52	*F*(000) = 472
Triclinic, *P*1	*D*_x_ = 1.284 Mg m^−^^3^
*a* = 9.5147 (5) Å	Mo *K*α_1_ radiation, λ = 0.71073 Å
*b* = 10.4479 (6) Å	Cell parameters from 9937 reflections
*c* = 11.4082 (6) Å	θ = 2.5–27.6°
α = 96.789 (2)°	µ = 0.10 mm^−^^1^
β = 93.287 (2)°	*T* = 115 K
γ = 90.945 (2)°	Prism, colourless
*V* = 1123.96 (11) Å^3^	0.5 × 0.3 × 0.25 mm

### Data collection

**Table:**

Nonius Kappa APEXII diffractometer	5190 independent reflections
Radiation source: X-ray tube, Siemens KFF Mo 2K-180	4273 reflections with *I* > 2σ(*I*)
Graphite monochromator	*R*_int_ = 0.030
φ and ω scans	θ_max_ = 27.6°, θ_min_ = 2.8°
Absorption correction: multi-scan (*SADABS*; Bruker, 2014)	*h* = −12→12
*T*_min_ = 0.710, *T*_max_ = 0.746	*k* = −13→13
30513 measured reflections	*l* = −14→14

### Refinement

**Table:**

Refinement on *F*^2^	0 restraints
Least-squares matrix: full	Hydrogen site location: inferred from neighbouring sites
*R*[*F*^2^ > 2σ(*F*^2^)] = 0.043	H-atom parameters constrained
*wR*(*F*^2^) = 0.115	*w* = 1/[σ^2^(*F*_o_^2^) + (0.0536*P*)^2^ + 0.724*P*] where *P* = (*F*_o_^2^ + 2*F*_c_^2^)/3
*S* = 1.03	(Δ/σ)_max_ < 0.001
5190 reflections	Δρ_max_ = 0.38 e Å^−^^3^
275 parameters	Δρ_min_ = −0.52 e Å^−^^3^

### Special details

**Table:**

Experimental. SADABS (Bruker, 2014) was used for absorption correction. wR2(int) was 0.0455 before and 0.0417 after correction. The ratio of minimum to maximum transmission is 0.9524. The λ/2 correction factor is 0.0015.
Geometry. All e.s.d.'s (except the e.s.d. in the dihedral angle between two l.s. planes) are estimated using the full covariance matrix. The cell e.s.d.'s are taken into account individually in the estimation of e.s.d.'s in distances, angles and torsion angles; correlations between e.s.d.'s in cell parameters are only used when they are defined by crystal symmetry. An approximate (isotropic) treatment of cell e.s.d.'s is used for estimating e.s.d.'s involving l.s. planes.

### Fractional atomic coordinates and isotropic or equivalent isotropic displacement parameters (Å^2^)

**Table:**

	*x*	*y*	*z*	*U*_iso_*/*U*_eq_	
C1	0.04814 (14)	0.86382 (12)	0.43156 (12)	0.0110 (3)	
C2	0.17149 (14)	0.86205 (13)	0.52171 (12)	0.0121 (3)	
H2D	0.1609	0.7851	0.5637	0.015*	
H2E	0.1672	0.9390	0.5809	0.015*	
C3	0.31605 (14)	0.86030 (13)	0.47093 (12)	0.0123 (3)	
H3A	0.3271	0.7759	0.4233	0.015*	
H3B	0.3203	0.9277	0.4170	0.015*	
C4	0.43892 (14)	0.88243 (12)	0.56279 (11)	0.0105 (3)	
C5	0.64831 (14)	1.09068 (14)	0.85914 (12)	0.0136 (3)	
C6	0.76641 (15)	1.09519 (14)	0.95460 (12)	0.0158 (3)	
H6A	0.7804	1.1849	0.9931	0.019*	
H6B	0.8548	1.0686	0.9180	0.019*	
C7	0.73497 (15)	1.00706 (14)	1.04801 (12)	0.0166 (3)	
H7A	0.6473	1.0346	1.0854	0.020*	
H7B	0.7193	0.9177	1.0092	0.020*	
C8	0.85386 (15)	1.00936 (14)	1.14279 (12)	0.0147 (3)	
C9	0.19496 (14)	0.31479 (13)	0.29397 (12)	0.0137 (3)	
H9	0.2923	0.3391	0.3279	0.016*	
C10	0.18667 (19)	0.33296 (15)	0.16317 (13)	0.0240 (3)	
H10A	0.0932	0.3024	0.1270	0.029*	
H10B	0.2592	0.2806	0.1225	0.029*	
C11	0.2096 (2)	0.47517 (16)	0.14622 (15)	0.0293 (4)	
H11A	0.3081	0.5016	0.1719	0.035*	
H11B	0.1955	0.4849	0.0611	0.035*	
C12	0.11074 (19)	0.56320 (15)	0.21517 (15)	0.0263 (4)	
H12A	0.0131	0.5463	0.1814	0.032*	
H12B	0.1362	0.6541	0.2081	0.036 (5)*	
C13	0.1185 (2)	0.54188 (15)	0.34496 (15)	0.0272 (4)	
H13A	0.0479	0.5956	0.3868	0.033*	
H13B	0.2129	0.5694	0.3811	0.039 (6)*	
C14	0.09117 (17)	0.40034 (14)	0.36036 (14)	0.0205 (3)	
H14A	0.1003	0.3893	0.4454	0.025*	
H14B	−0.0060	0.3745	0.3302	0.025*	
C15	0.31417 (15)	0.36301 (13)	0.71133 (12)	0.0143 (3)	
H15	0.2123	0.3698	0.6867	0.017*	
C16	0.40040 (18)	0.43583 (14)	0.63179 (14)	0.0208 (3)	
H16A	0.3808	0.3989	0.5483	0.025*	
H16B	0.5020	0.4267	0.6523	0.025*	
C17	0.3636 (2)	0.57889 (15)	0.64653 (15)	0.0275 (4)	
H17A	0.4231	0.6258	0.5966	0.033*	
H17B	0.2640	0.5880	0.6191	0.033*	
C18	0.38570 (19)	0.63880 (15)	0.77466 (15)	0.0264 (4)	
H18A	0.3541	0.7291	0.7817	0.032*	
H18B	0.4873	0.6399	0.7990	0.032*	
C19	0.30447 (19)	0.56390 (15)	0.85661 (14)	0.0245 (3)	
H19A	0.2023	0.5748	0.8406	0.029*	
H19B	0.3290	0.5998	0.9397	0.029*	
C20	0.33692 (17)	0.41964 (14)	0.84057 (13)	0.0196 (3)	
H20A	0.4358	0.4075	0.8682	0.024*	
H20B	0.2751	0.3734	0.8893	0.024*	
N1	0.16561 (12)	0.17671 (11)	0.30888 (10)	0.0136 (2)	
H1A	0.2281	0.1259	0.2689	0.020*	
H1B	0.1743	0.1663	0.3870	0.020*	
H1C	0.0765	0.1539	0.2798	0.020*	
N2	0.35079 (12)	0.22343 (11)	0.69782 (10)	0.0130 (2)	
H2A	0.2985	0.1808	0.7459	0.020*	
H2B	0.3322	0.1890	0.6213	0.020*	
H2C	0.4439	0.2157	0.7181	0.020*	
O1	0.07729 (10)	0.90365 (10)	0.33203 (8)	0.0147 (2)	
H1	0.0031	0.9042	0.2885	0.022*	
O2	−0.07178 (10)	0.83211 (10)	0.45502 (9)	0.0148 (2)	
O3	0.55754 (10)	0.84347 (9)	0.54021 (8)	0.0145 (2)	
O4	0.41065 (10)	0.94748 (10)	0.66222 (8)	0.0139 (2)	
H4	0.4842	0.9578	0.7069	0.021*	
O5	0.61844 (10)	0.97769 (10)	0.80258 (9)	0.0159 (2)	
O6	0.58720 (12)	1.19027 (11)	0.84010 (10)	0.0241 (3)	
O7	0.93003 (13)	1.10688 (11)	1.16655 (11)	0.0292 (3)	
O8	0.86738 (10)	0.90635 (10)	1.19411 (9)	0.0159 (2)	

### Atomic displacement parameters (Å^2^)

**Table:**

	*U*^11^	*U*^22^	*U*^33^	*U*^12^	*U*^13^	*U*^23^
C1	0.0143 (6)	0.0071 (6)	0.0113 (6)	0.0018 (5)	−0.0007 (5)	0.0001 (5)
C2	0.0118 (6)	0.0142 (6)	0.0105 (6)	0.0015 (5)	−0.0020 (5)	0.0029 (5)
C3	0.0121 (6)	0.0137 (6)	0.0105 (6)	−0.0015 (5)	−0.0017 (5)	−0.0002 (5)
C4	0.0130 (6)	0.0074 (6)	0.0113 (6)	−0.0017 (5)	−0.0006 (5)	0.0031 (5)
C5	0.0138 (6)	0.0176 (7)	0.0093 (6)	0.0000 (5)	−0.0018 (5)	0.0017 (5)
C6	0.0165 (7)	0.0185 (7)	0.0119 (6)	−0.0021 (5)	−0.0059 (5)	0.0031 (5)
C7	0.0166 (7)	0.0190 (7)	0.0138 (7)	−0.0046 (5)	−0.0072 (5)	0.0048 (6)
C8	0.0153 (7)	0.0176 (7)	0.0111 (6)	−0.0014 (5)	−0.0031 (5)	0.0032 (5)
C9	0.0147 (6)	0.0109 (6)	0.0149 (7)	−0.0021 (5)	0.0003 (5)	0.0001 (5)
C10	0.0407 (9)	0.0163 (7)	0.0154 (7)	0.0000 (7)	0.0091 (7)	0.0005 (6)
C11	0.0479 (11)	0.0200 (8)	0.0221 (8)	−0.0010 (7)	0.0161 (8)	0.0054 (6)
C12	0.0346 (9)	0.0148 (7)	0.0316 (9)	0.0006 (6)	0.0066 (7)	0.0092 (6)
C13	0.0420 (10)	0.0125 (7)	0.0282 (9)	0.0022 (7)	0.0164 (7)	0.0003 (6)
C14	0.0277 (8)	0.0149 (7)	0.0202 (7)	0.0020 (6)	0.0111 (6)	0.0032 (6)
C15	0.0166 (7)	0.0105 (6)	0.0160 (7)	0.0019 (5)	0.0013 (5)	0.0020 (5)
C16	0.0311 (8)	0.0145 (7)	0.0183 (7)	0.0034 (6)	0.0094 (6)	0.0039 (6)
C17	0.0462 (10)	0.0143 (7)	0.0253 (8)	0.0067 (7)	0.0153 (7)	0.0089 (6)
C18	0.0382 (9)	0.0114 (7)	0.0307 (9)	−0.0004 (6)	0.0132 (7)	0.0016 (6)
C19	0.0376 (9)	0.0140 (7)	0.0222 (8)	0.0007 (6)	0.0119 (7)	−0.0007 (6)
C20	0.0316 (8)	0.0128 (7)	0.0150 (7)	−0.0003 (6)	0.0061 (6)	0.0017 (5)
N1	0.0130 (5)	0.0120 (6)	0.0153 (6)	−0.0004 (4)	−0.0031 (4)	0.0018 (4)
N2	0.0138 (6)	0.0105 (5)	0.0142 (6)	−0.0004 (4)	−0.0020 (4)	0.0004 (4)
O1	0.0121 (5)	0.0206 (5)	0.0120 (5)	−0.0005 (4)	−0.0039 (4)	0.0060 (4)
O2	0.0121 (5)	0.0173 (5)	0.0153 (5)	−0.0013 (4)	−0.0007 (4)	0.0039 (4)
O3	0.0123 (5)	0.0159 (5)	0.0146 (5)	0.0019 (4)	−0.0007 (4)	−0.0001 (4)
O4	0.0119 (5)	0.0177 (5)	0.0108 (5)	0.0013 (4)	−0.0039 (4)	−0.0014 (4)
O5	0.0162 (5)	0.0163 (5)	0.0138 (5)	0.0011 (4)	−0.0053 (4)	−0.0008 (4)
O6	0.0271 (6)	0.0190 (5)	0.0241 (6)	0.0059 (4)	−0.0117 (5)	−0.0001 (4)
O7	0.0322 (6)	0.0232 (6)	0.0313 (6)	−0.0134 (5)	−0.0216 (5)	0.0127 (5)
O8	0.0155 (5)	0.0179 (5)	0.0147 (5)	−0.0013 (4)	−0.0046 (4)	0.0064 (4)

### Geometric parameters (Å, º)

**Table:**

C1—C2	1.5174 (18)	C12—H12B	0.9900
C1—O1	1.2974 (17)	C12—C13	1.521 (2)
C1—O2	1.2356 (17)	C13—H13A	0.9900
C2—H2D	0.9900	C13—H13B	0.9900
C2—H2E	0.9900	C13—C14	1.530 (2)
C2—C3	1.5224 (19)	C14—H14A	0.9900
C3—H3A	0.9900	C14—H14B	0.9900
C3—H3B	0.9900	C15—H15	1.0000
C3—C4	1.5204 (18)	C15—C16	1.518 (2)
C4—O3	1.2367 (17)	C15—C20	1.524 (2)
C4—O4	1.2961 (16)	C15—N2	1.4972 (17)
C5—C6	1.5155 (18)	C16—H16A	0.9900
C5—O5	1.2955 (17)	C16—H16B	0.9900
C5—O6	1.2356 (18)	C16—C17	1.533 (2)
C6—H6A	0.9900	C17—H17A	0.9900
C6—H6B	0.9900	C17—H17B	0.9900
C6—C7	1.527 (2)	C17—C18	1.522 (2)
C7—H7A	0.9900	C18—H18A	0.9900
C7—H7B	0.9900	C18—H18B	0.9900
C7—C8	1.5172 (18)	C18—C19	1.523 (2)
C8—O7	1.2348 (18)	C19—H19A	0.9900
C8—O8	1.2894 (17)	C19—H19B	0.9900
C9—H9	1.0000	C19—C20	1.535 (2)
C9—C10	1.524 (2)	C20—H20A	0.9900
C9—C14	1.517 (2)	C20—H20B	0.9900
C9—N1	1.4961 (17)	N1—H1A	0.9100
C10—H10A	0.9900	N1—H1B	0.9100
C10—H10B	0.9900	N1—H1C	0.9100
C10—C11	1.534 (2)	N2—H2A	0.9100
C11—H11A	0.9900	N2—H2B	0.9100
C11—H11B	0.9900	N2—H2C	0.9100
C11—C12	1.514 (2)	O1—H1	0.8400
C12—H12A	0.9900	O4—H4	0.8400
			
O1—C1—C2	115.61 (11)	C12—C13—H13B	109.3
O2—C1—C2	120.85 (12)	C12—C13—C14	111.59 (13)
O2—C1—O1	123.51 (12)	H13A—C13—H13B	108.0
C1—C2—H2D	108.5	C14—C13—H13A	109.3
C1—C2—H2E	108.5	C14—C13—H13B	109.3
C1—C2—C3	115.06 (11)	C9—C14—C13	110.61 (12)
H2D—C2—H2E	107.5	C9—C14—H14A	109.5
C3—C2—H2D	108.5	C9—C14—H14B	109.5
C3—C2—H2E	108.5	C13—C14—H14A	109.5
C2—C3—H3A	108.6	C13—C14—H14B	109.5
C2—C3—H3B	108.6	H14A—C14—H14B	108.1
H3A—C3—H3B	107.6	C16—C15—H15	108.4
C4—C3—C2	114.67 (11)	C16—C15—C20	111.50 (12)
C4—C3—H3A	108.6	C20—C15—H15	108.4
C4—C3—H3B	108.6	N2—C15—H15	108.4
O3—C4—C3	120.91 (12)	N2—C15—C16	110.23 (11)
O3—C4—O4	123.68 (12)	N2—C15—C20	109.86 (11)
O4—C4—C3	115.37 (11)	C15—C16—H16A	109.6
O5—C5—C6	115.35 (12)	C15—C16—H16B	109.6
O6—C5—C6	120.21 (13)	C15—C16—C17	110.12 (12)
O6—C5—O5	124.44 (12)	H16A—C16—H16B	108.2
C5—C6—H6A	109.2	C17—C16—H16A	109.6
C5—C6—H6B	109.2	C17—C16—H16B	109.6
C5—C6—C7	111.84 (12)	C16—C17—H17A	109.3
H6A—C6—H6B	107.9	C16—C17—H17B	109.3
C7—C6—H6A	109.2	H17A—C17—H17B	107.9
C7—C6—H6B	109.2	C18—C17—C16	111.69 (13)
C6—C7—H7A	109.2	C18—C17—H17A	109.3
C6—C7—H7B	109.2	C18—C17—H17B	109.3
H7A—C7—H7B	107.9	C17—C18—H18A	109.4
C8—C7—C6	112.13 (12)	C17—C18—H18B	109.4
C8—C7—H7A	109.2	C17—C18—C19	111.36 (14)
C8—C7—H7B	109.2	H18A—C18—H18B	108.0
O7—C8—C7	119.57 (13)	C19—C18—H18A	109.4
O7—C8—O8	124.26 (13)	C19—C18—H18B	109.4
O8—C8—C7	116.16 (12)	C18—C19—H19A	109.2
C10—C9—H9	108.7	C18—C19—H19B	109.2
C14—C9—H9	108.7	C18—C19—C20	112.09 (13)
C14—C9—C10	110.73 (12)	H19A—C19—H19B	107.9
N1—C9—H9	108.7	C20—C19—H19A	109.2
N1—C9—C10	110.22 (11)	C20—C19—H19B	109.2
N1—C9—C14	109.86 (11)	C15—C20—C19	111.06 (12)
C9—C10—H10A	109.4	C15—C20—H20A	109.4
C9—C10—H10B	109.4	C15—C20—H20B	109.4
C9—C10—C11	111.02 (13)	C19—C20—H20A	109.4
H10A—C10—H10B	108.0	C19—C20—H20B	109.4
C11—C10—H10A	109.4	H20A—C20—H20B	108.0
C11—C10—H10B	109.4	C9—N1—H1A	109.5
C10—C11—H11A	109.1	C9—N1—H1B	109.5
C10—C11—H11B	109.1	C9—N1—H1C	109.5
H11A—C11—H11B	107.8	H1A—N1—H1B	109.5
C12—C11—C10	112.56 (13)	H1A—N1—H1C	109.5
C12—C11—H11A	109.1	H1B—N1—H1C	109.5
C12—C11—H11B	109.1	C15—N2—H2A	109.5
C11—C12—H12A	109.5	C15—N2—H2B	109.5
C11—C12—H12B	109.5	C15—N2—H2C	109.5
C11—C12—C13	110.91 (14)	H2A—N2—H2B	109.5
H12A—C12—H12B	108.0	H2A—N2—H2C	109.5
C13—C12—H12A	109.5	H2B—N2—H2C	109.5
C13—C12—H12B	109.5	C1—O1—H1	109.5
C12—C13—H13A	109.3	C4—O4—H4	109.5
			
C1—C2—C3—C4	169.67 (11)	C16—C15—C20—C19	55.77 (17)
C2—C3—C4—O3	156.32 (12)	C16—C17—C18—C19	−54.8 (2)
C2—C3—C4—O4	−25.91 (16)	C17—C18—C19—C20	53.0 (2)
C5—C6—C7—C8	179.04 (12)	C18—C19—C20—C15	−53.40 (19)
C6—C7—C8—O7	28.2 (2)	C20—C15—C16—C17	−57.25 (17)
C6—C7—C8—O8	−152.14 (13)	N1—C9—C10—C11	177.34 (13)
C9—C10—C11—C12	−54.0 (2)	N1—C9—C14—C13	−179.35 (13)
C10—C9—C14—C13	−57.37 (17)	N2—C15—C16—C17	−179.55 (13)
C10—C11—C12—C13	53.3 (2)	N2—C15—C20—C19	178.28 (12)
C11—C12—C13—C14	−54.75 (19)	O1—C1—C2—C3	−20.75 (17)
C12—C13—C14—C9	57.21 (19)	O2—C1—C2—C3	161.07 (12)
C14—C9—C10—C11	55.57 (18)	O5—C5—C6—C7	−58.22 (17)
C15—C16—C17—C18	56.77 (19)	O6—C5—C6—C7	121.41 (15)

### Hydrogen-bond geometry (Å, º)

**Table:**

*D*—H···*A*	*D*—H	H···*A*	*D*···*A*	*D*—H···*A*
N1—H1*A*···O5^i^	0.91	1.99	2.8923 (16)	173
N1—H1*B*···O2^ii^	0.91	2.10	2.8969 (16)	146
N1—H1*C*···O7^iii^	0.91	1.86	2.7279 (15)	158
N2—H2*A*···O8^iv^	0.91	2.00	2.8746 (16)	160
N2—H2*B*···O3^i^	0.91	2.17	2.9098 (15)	138
N2—H2*C*···O6^v^	0.91	1.94	2.7485 (15)	148
O1—H1···O8^vi^	0.84	1.64	2.4734 (13)	175
O4—H4···O5	0.84	1.63	2.4636 (13)	175

Symmetry codes: (i) −*x*+1, −*y*+1, −*z*+1; (ii) −*x*, −*y*+1, −*z*+1; (iii) *x*−1, *y*−1, *z*−1; (iv) −*x*+1, −*y*+1, −*z*+2; (v) *x*, *y*−1, *z*; (vi) *x*−1, *y*, *z*−1.
